# Psychiatric presentations in acute illness with COVID-19: a retrospective analysis

**DOI:** 10.1192/bjo.2021.168

**Published:** 2021-06-18

**Authors:** Elizabeth Nelmes, Audrey Ng

**Affiliations:** West London NHS Trust

## Abstract

**Aims:**

To assess the psychiatric presentations in patients with a diagnosis of COVID-19 referred to a liaison psychiatry department during a one month period in the peak of the global pandemic.

**Method:**

A retrospective analysis of the patients referred to liaison psychiatry during January 2021 who also had a diagnosis of COVID-19. Confirmed cases of COVID-19 were defined as those confirmed by COVID-19 PCR in respiratory samples or clinically suspected cases from chest radiograph or CT. Severe COVID-19 was defined as those requiring supplementary oxygen due to saturations of 93% or less.

**Result:**

During January 2021, a total of 24 patients were referred to liaison psychiatry with concurrent COVID-19 infection. Out of these patients, 63% had a previous mental health diagnosis. The most common reason for referral was low mood (37.5%), followed by agitation (25%) and psychosis (25%). When considering first psychiatric presentations with concurrent COVID-19 infection, the most common presentation was psychosis (44%). The time course of psychosis was most frequently seen in the seven days prior to a positive swab. In one case a patient was sectioned under the Mental Health Act for psychosis two days prior to developing symptoms. Two of these patients were worked up for possible encephalitis including radiological imaging and lumbar puncture. For patients defined as having severe COVID-19, the most common referral was low mood. In those referred for low mood, 66% had a history of an affective disorder. In two cases low mood was complicated by an acute stress reaction to recent bereavement. For one patient this included the bereavement of two relatives to COVID-19. For patients admitted to intensive care and intubated for respiratory support the most common referrals were low mood and agitation. These factors we found a barrier to successful rehabilitation following periods of significant illness.

**Conclusion:**

The impact of COVID-19 on psychiatric presentations extends beyond the socio-economic factors precipitating crises across the nation. Our findings of acute psychiatric illness in the prodromal phase of the viral illness suggest a neuropsychiatric pathogenesis to COVID-19.

